# The Effects of Probiotic Soymilk Fortified with Omega-3 on Blood Glucose, Lipid Profile, Haematological and Oxidative Stress, and Inflammatory Parameters in Streptozotocin Nicotinamide-Induced Diabetic Rats

**DOI:** 10.1155/2015/696372

**Published:** 2015-08-11

**Authors:** Mohsen Mohammadi Sartang, Seyed Mohammad Mazloomi, Nader Tanideh, Abbas Rezaian Zadeh

**Affiliations:** ^1^Student Research Committee, School of Nutrition and Food Sciences, Shiraz University of Medical Sciences, Shiraz 7153675541, Iran; ^2^Department of Food Hygiene and Quality Control, Nutrition and Food Sciences Research Center, School of Nutrition and Food Sciences, Shiraz University of Medical Sciences, Shiraz 7153675541, Iran; ^3^Stem Cell and Transgenic Technology Research Center, Department of Pharmacology, School of Medicine, Shiraz University of Medical Sciences, Shiraz 7134874478, Iran; ^4^Research Center for Health Sciences, Department of Epidemiology, School of Health, Shiraz University of Medical Sciences, Shiraz 7153675541, Iran

## Abstract

*Objective*. The aim of the present study was to evaluate the effects of probiotic soymilk fortified with omega-3 in diabetic rats. *Methods*. Soymilk (SM), fermented soymilk (FSM), and fermented soymilk fortified with omega-3 (FSM + omega-3) were prepared. Rats were randomly assigned to five groups of 13 animals per group. Diabetes was induced by a single injection of streptozotocin (STZ) 15 min after the intraperitoneal administration of nicotinamide (NA). Normal control (NC) and diabetic control (DC) rats received 1 mL/day of distilled water and three groups of diabetic rats were given 1 mL/day of SM, FSM, and FSM + omega-3 products by oral gavage for 28 days. *Results*. Three products significantly (*P* < 0.05) reduced blood glucose, total cholesterol (TC), triglyceride (TG), and malondialdehyde (MDA) concentrations compared to the DC group, with the maximum reduction seen in the FSM + omega-3 group. Body weight, red blood cells (RBC), haemoglobin (Hb), haematocrit, and superoxide dismutase (SOD) also significantly increased in the FSM + omega-3 group. In the FSM + omega-3 group, MDA level compared with the SM and FSM groups and high sensitivity C-reactive protein (hs-CRP) concentrations compared with the DC and FSM groups were significantly lower (*P* < 0.05). *Conclusion*. Fermented soymilk fortified with omega-3 may be beneficial in diabetes.

## 1. Introduction

Diabetes mellitus is a largely occurring endocrine disorder in many countries [1]. In diabetes, due to defects in the production of insulin or its action, blood glucose levels become elevated. Also impaired is the functioning of the macronutrient metabolism, leading to long-term health complications [2]. In addition, free radicals generated during long-term hyperglycaemia impair the body's antioxidant defence system [3]. Diabetes treatment is based on pharmacological hypoglycaemic agents and insulin; however, the efficacy of these therapies is limited due to their many side effects. Therefore, finding natural compounds is essential for overcoming these problems [4, 5].

According to epidemiological studies, trends toward the use of soy products are growing, since soy consumption is associated with a decrease in certain diseases, including diabetes and atherosclerosis [4]. Soymilk contains high-quality proteins, dietary fibre, small quantities of saturated fatty acids, and no cholesterol and lactose, which make it suitable for people who are lactose intolerant. The antidiabetic and antiobesity effects of soymilk have also been highlighted [6]. However, soy consumption remains limited due to factors such as its taste and oligosaccharides such as raffinose and stachyose, which often leads to bloating and stomach discomfort [7]. Fermentation is a suitable method for improving the properties of isoflavones and peptides in soybean and this process increases the efficacy of these components in the treatment or prevention of type 2 diabetes [8]. In fact, isoflavone glycosides are changed into isoflavone aglycones following fermentation, which seem to have greater physiological effects and are better absorbed [9].

Probiotic food containing bifidobacteria results in the decreased total cholesterol and LDL-C and increased HDL-C [10]. Therefore, fermentation of food products by these probiotic bacteria was increased [11]. In addition, sugars in soymilk are suitable nutrients for bifidobacteria growth [12].

The beneficial effect of fermentation is not only that it increases the bioavailability of isoflavones, but also that it aids in the digestion of protein, the solubility of calcium, and the enhancement of intestinal health and the immune system [6]. The beneficial effects of soymilk fermented with* Bifidobacterium* on lipid profiles have been indicated in previous studies [13]. Essential fatty acid levels in various tissues have been reduced in diabetes through the use of soymilk. This may be due to a reduction in the conversion of linolenic acid to EPA and DHA. Supplementation with omega-3 fatty acids in patients with diabetes has attracted significant attention and may be effective at reducing some of the complications associated with diabetes. However, due to their double bonds, these fatty acids are susceptible to oxidation and may thus increase oxidative stress [14]. Human and animal studies have shown conflicting results regarding the effect of supplementation with fish oil omega-3 fatty acids in terms of oxidant/antioxidant status [15, 16].

Increased consumption of omega-3 fatty acids due to the adverse effects of inadequate intake is recommended. The fortification of food products, without extensive changes to eating habits, is a suitable method for increasing omega-3 content in the diet. Furthermore, this fatty acid can be used for potentiating probiotic effects in the small intestine by changing fatty acid composition [17].

The present study therefore aimed to determine whether combining soymilk, probiotics, and omega-3 had additional effects on blood glucose, lipid profiles, haematological and oxidative stress, and the inflammatory parameters within a diabetic type 2 animal model.

## 2. Methods

### 2.1. Preparation of Products

Soymilk was prepared according to the method described by previous studies [18]. Initially, after soaking soybeans in distilled water overnight, the water was discarded and the soaked soybeans were combined with distilled water 10 times their weight, and the mixture was mixed in a blender for three minutes. Then, the mixture was passed through a filter to produce soymilk. The soymilk was then divided into three equal parts. All samples were sterilized at 121°C for 15 min and cooled to 37°C. One part was used as the control sample and 0.1 g/L of* Bifidobacterium lactis* (Christin Hansen, Denmark) was added to the other two samples. Omega-3 (SERVA, Feinbiochemica, Heidelberg, New York, USA) (1 g/L) was also added to one sample. Samples were inoculated at 37°C until their pH reached 4.7. The samples were then stored in a refrigerator. The fermented soymilk samples were prepared once a week under hygienic conditions.

### 2.2. Experimental Animals

At the beginning of the experiment, 65 male Sprague-Dawley rats (weighing 200–300 g each) were purchased from the Laboratory Animals Research Center (Shiraz University of Medical Sciences, Iran). The animals were acclimatized to the laboratory for two weeks prior to starting the experiments and were fed a chow diet (Pars Dam Co., Tehran, Iran) and regular drinking water ad libitum during the study; rats were kept in stainless steel cages in groups of three animals per cage in a temperature-controlled (22–25°C) environment; lighting (12 hr light/dark cycles) and humidity (%50 ± 5) conditions were also controlled. Animal procedures in our study were carried out according to ethics stated in the Guide for the Care and Use of Laboratory Animals [19].

### 2.3. Induction of Diabetes

In the present study, type 2 diabetes was induced intraperitoneally (IP) in the overnight-fasted male Sprague-Dawley rats through the injection of freshly prepared streptozotocin (STZ) (65 mg/kg body weight; Sigma, USA), dissolved in a 0.1 mol/L citrate buffer (pH 4.5), 15 min following the IP administration of nicotinamide (NA) (110 mg/kg body weight; Merck, Germany) dissolved in normal saline [20]. A glucometer (Accu-Chek Active, Roche, Germany) was used for the estimation of blood glucose levels. The stable blood glucose concentration seven days after STZ-NA injection was used for the confirmation of diabetes. Blood glucose levels above 150 mg dL were considered as criteria for diabetes.

### 2.4. Experimental Design

Diabetic rats were divided randomly into four groups of 13 rats per group. One group was also considered as normal control rats. The treatment period for the study was 28 days. Products were administered to rats by oral gavage at a level of 1 mL/day.

*Group I* included normal control (NC) rats given 1 mL of distilled water;* Group II* included diabetic control (DC) rats given 1 mL of distilled water;* Group III* included diabetic rats given 1 mL/day of soymilk (SM);* Group IV* included diabetic rats given 1 mL/day of fermented soymilk (FSM);* Group V* included diabetic rats given 1 mL/day of fermented soymilk fortified with omega-3 (FSM + omega-3).

### 2.5. Determination of Biochemical Parameters

Rats were monitored weekly regarding body weight and blood glucose. On day 29, the rats were fasted for 12 hours and under anaesthesia (50 mg/kg ketamine plus 5 mg/kg diazepam administered intraperitoneally), approximately 5 mL of blood was collected by cardiac puncture, 1 mL collected into a tube containing EDTA for measuring haematological parameters, and the remaining centrifuged at 3500 rpm for 10 min for the separation of serum. Each serum sample was stored in clean sterile microcentrifuge tubes at −80°C until analysis.

### 2.6. Analytical Measurements

The serum levels of total cholesterol (TC), triglyceride (TG), HDL cholesterol (HDL-C), and LDL cholesterol (LDL-C) were assayed for each rat with the aid of specific enzyme kits (Pars Azmoon Co., Tehran, Iran), which were used according to the manufacturer's instructions. A cell counter was used to measure haematological parameters. The malondialdehyde (MDA) level of serum was determined using the thiobarbituric acid (TBARS) colorimetric analysis method. Optical density was determined at 532 nm [21]. A commercial kit (Ransod, Randox Laboratories Ltd.) was used for the measuring of superoxide dismutase (SOD) activities in erythrocyte according to manufacturer's instructions. Serum high-sensitive C-reactive protein (hs-CRP) concentrations were measured using the ELISA kit according to the manufacturer's instructions.

### 2.7. Statistical Analysis

The data were represented as mean ± standard deviation (SD). The statistical analysis was performed using SPSS (version 19.0). One-way repeated-measures analysis of variance (ANOVA) was used to compare the mean blood glucose and body weight between groups at different measurement times. For other parameters at the end of the experiment, a one-way analysis of variance (ANOVA) procedure was used, followed by post hoc Duncan's multiple range tests.

## 3. Results

### 3.1. Blood Glucose

Results of ANOVA with repeated measures showed significant changes in the blood glucose levels of diabetic rats from the first week until the end of the experiment; however, the trend of decreasing blood glucose was different among the five groups (Table 1). NC Rats (Group I) maintained a normal blood glucose level during the study. The IP administration of STZ-NA to rats significantly increased the level of blood sugar compared to NC rats. Blood glucose level was increased from 114.08 to 162.58 mg/dl seven days following STZ-NA administration. At the end of experiment, blood glucose in all treated diabetic rats had been significantly reduced (*P* < 0.05) compared to the DC rats. FSM + omega-3, FSM and SM exhibited the greatest reduction –47.2%, 39.3% and 35.8%, respectively (Table 1).

### 3.2. Body Weight

Results of ANOVA with repeated measures showed significant changes in the body weight of diabetic rats from the first week until the end of the experiments (Table 2). There was no significant difference in the initial body weight among the five groups (*P* > 0.05). Diabetic rats showed a significant decrease in body weight compared to the control group (*P* < 0.05). Oral administration of three different products (SM, FSM and FSM + omega-3 ) for 28 days improved body weight significantly (*P* < 0.05) with the maximum weight gain seen in the FSM + omega-3 group compared to other groups (Table 2).

### 3.3. Serum Lipids

The effects of treatments on serum lipids are shown in Table 3. TC and TG concentrations of all treated diabetic rats were significantly decreased (*P* < 0.05) compared to the DC group, with maximum reduction seen in the FSM + omega-3 group (20.8% for TC and 39.3% for TG). The FSM + omega-3 product also had a tendency to produce lower TG concentrations than FSM and SM products (6.2% relative to SM and 11.2% relative to FSM). Although no differences in HDL-C and LDL-C concentrations among the four diabetic groups were observed, when compared with the DC group, the FSM product had a tendency to produce greater HDL-C concentrations compared to the SM and FSM + omega-3 groups (24.8%, 20.4%, and 14.8%, resp.). According to statistical comparisons between treated groups and the NC group, there was no significant difference between SM, FSM, and FSM + omega-3 groups in terms of lowering LDL-C level (Table 3).

### 3.4. Haematological Parameters

The effects of treatments on haematological parameters are shown in Table 4. Haemoglobin (Hb), mean corpuscular haemoglobin (MCH), and mean corpuscular haemoglobin concentration (MCHC) in the diabetic control group were significantly decreased compared to the NC group (*P* < 0.05). Hb, red blood cells (RBC), and haematocrit of the SM, FSM, and FSM + omega-3 groups and mean corpuscular volume (MCV) and MCH for the SM and FSM + omega-3 were significantly increased (*P* < 0.05) compared with the DC group, with the greatest increase seen in the FSM + omega-3 group (Table 4).

### 3.5. Oxidative Stress and Inflammatory Parameters

**Figure 1:**
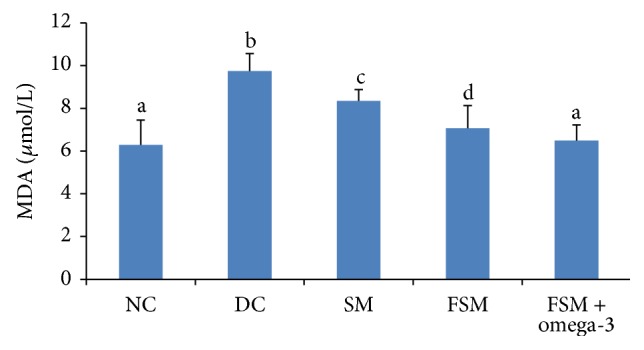
MDA level within the five groups. NC = normal control, DC = diabetic control, SM = diabetic rats that received soymilk, FSM = diabetic rats that received fermented soymilk, and FSM + omega-3 = diabetic rats that received fermented soymilk fortified with omega-3. Each value is expressed as mean ± SD. Values with different letters are significantly different at* P* < 0.05 as analyzed by Duncan's multiple range test.

**Figure 2:**
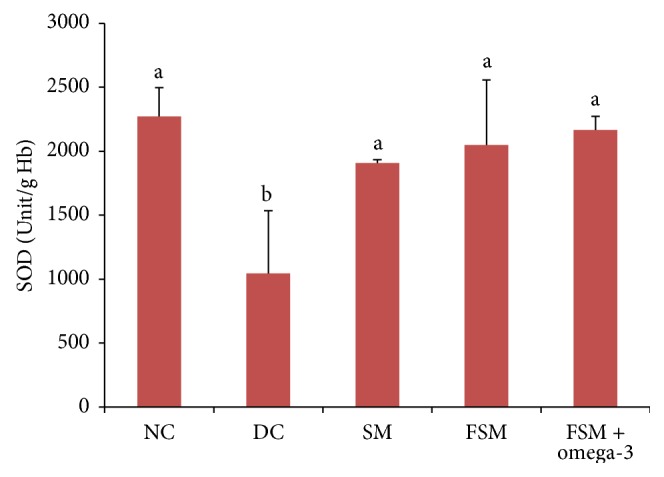
SOD concentrations within the five groups. NC = normal control, DC = diabetic control, SM = diabetic rats that received soymilk, FSM = diabetic rats that received fermented soymilk, and FSM + omega-3 = diabetic rats that received fermented soymilk fortified with omega-3. Each value is expressed as mean ± SD. Values with different letters are significantly different at *P* < 0.05 as analyzed by Duncan's multiple range test.

**Figure 3:**
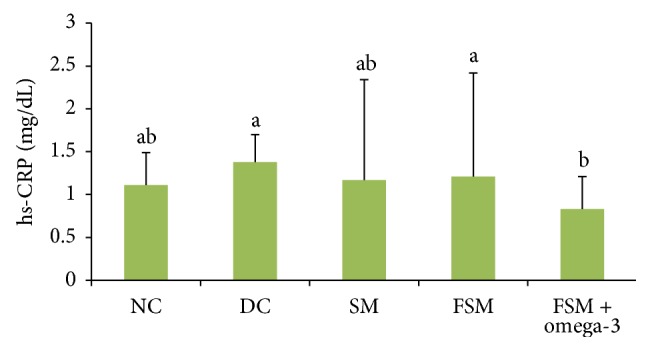
hs-CRP concentrations within the five groups. NC = normal control, DC = diabetic control, SM = diabetic rats that received soymilk, FSM = diabetic rats that received fermented soymilk, and FSM + omega-3 = diabetic rats that received fermented soymilk fortified with omega-3. Each value is expressed as mean ± SD. Values with different letters are significantly different at *P* < 0.05 as analyzed by Duncan's multiple range test.

The effects of treatments are shown in Figures 1, 2, and 3. SOD concentration was decreased, but MDA had been significantly increased in the DC group compared to the NC group (*P* < 0.001). By the end of experiment, there was no significant difference in high-sensitive C-reactive protein (hs-CRP) concentrations between these two groups. FSM + omega-3, FSM, and SM groups showed significantly higher SOD concentrations (107.3%, 95.9%, and 82.5%, resp.) when compared with the DC group (*P* < 0.05), with maximum concentrations observed in the FSM + omega-3 group. Additionally, all treated diabetic rats showed significantly (*P* < 0.05) lower MDA concentrations than the DC group (*P* < 0.001), with the greatest reduction concentrations observed in the FSM + omega-3 group (33.4% versus 14.3% for SM and 27.4% for FSM). MDA concentrations in the FSM + omega-3 group had been significantly decreased (*P* < 0.05) compared to the SM and FSM group; hs-CRP concentrations in the FSM + omega-3 group were significantly decreased compared to those of the DC and FSM group (*P* < 0.05).

## 4. Discussion

In our study, experimentally induced diabetes significantly increased blood glucose levels by 197% higher than that of the control level. Uptake and utilization of glucose, as well as glucose metabolism, are disturbed in diabetes mellitus [8]. For evaluating the hypoglycaemic effects of different compounds, the STZ-NA model appears to be a better model than its STZ counterpart, because the former manifests only a mild hyperglycaemic state [20].

The glucose level (≥150 mg/dL) recorded in rats underscored the real diabetic status of the rats in our study. The treatment of diabetic rats with SM, FSM, and FSM + omega-3 products in our study had after 28 days significantly reduced blood glucose levels by 35.8%, 39.3%, and 47.2%, respectively, compared to the DC group.

It appeared that the hypoglycaemic effects of these products observed in our study were due to soy protein and isoflavones present in soymilk, as the hypoglycaemic effects of these components have been reported in previous studies [22]. Isoflavonoids and protein in soybean are connected to reducing insulin resistance and improving glycaemic control [23], although these results differ from some other published studies [24, 25]. The antidiabetic actions of isoflavonoids may potentially be exerted via oestrogen receptors and for this reason this activity of soy isoflavones is beneficial for improving glucose metabolism [26]. Diabetic rats that received FSM were found to have reduced plasma glucose levels, more so than SM. The effects of fermented soymilk on glucose status have previously been evaluated [27]. In 2005, Kawakami et al. demonstrated that isoflavonoid glucoside that changed into aglycones following fermentation had better activity than isoflavonoid glycones and that the intake of isoflavones aglycones significantly increased serum isoflavone concentration, compared to isoflavone glycoside [27]. Thus, in our study, the additional effect of fermented soymilk for controlling the glucose metabolism may have been due to an increase in isoflavonoid aglycones. We also observed that FSM + omega-3 decreased blood glucose, more so than SM and FSM. In 1989, Linn et al. showed that an increasing intake of omega-3 fatty acids can reduce hyperglycaemia, as well as the risk of diabetes in rats [28].

It was observed that the intake of SM, FSM, and FSM + omega-3 products results in an increase in body weight when compared with the DC group. Soybean isoflavones improve metabolism in the presence of diabetes, resulting not only in suppressing weight loss, but also in a weight increase [29].

In this study, TG and TC concentrations were significantly increased in the DC group compared to concentrations in the NC group. These findings were in agreement with other findings [30] that have shown that a plasma increase in these parameters was frequently observed in diabetes mellitus states.

In the present study, we observed that the TC and TG concentrations of the SM, FSM, and FSM + omega-3 groups had been significantly decreased compared to those of the DC group. Our findings regarding the hypocholesterolaemic effects of soymilk and* Bifidobacterium* soymilk are in agreement with Kikuchi-Hayakawa's results [9], which showed that fermented soymilk suppresses cholesterol synthesis in rats. Moreover, the significant effect of fermented soymilk with* Bifidobacterium* on TC in rats has also been demonstrated in other studies [13, 31]. It could be assessed that not only the protein but also isoflavones in soymilk reduce the concentrations of serum lipids [32] and can be seen as having antiatherogenic effects [33].

With regard to the lowering TC and TG concentrations, it can be argued that soymilk may act according to its components such as isoflavones and soy protein by (1) decreasing insulin to glucagon ratio and finally reducing the expression of lipogenic genes [34]; (2) activating PPAR-*γ* (peroxisome proliferator-activated receptor gamma), with upregulation of adipogenesis [35]; (3) and binding of isoflavone to oestrogen receptors [36]. In addition, amino acids, minerals, and phytic acid, as well as other soy bioactive components in soymilk are effective in the decrease of TC and TG [37].

With regard to the effects on HDL-C levels, feeding SM, FSM, and FSM + omega-3 products to the DC group resulted in a higher HDL-C level, but this difference was not significant. The FSM product also had a tendency to produce greater HDL-C than SM and FSM + omega-3 products. These findings support the ideas of Rossi et al. (2000), who suggested that soy fermented products cause a decrease in TC and an increase in HDL-C concentrations [38].

These beneficial effects of FSM + omega-3 on suppressing an increase in plasma TG levels were greater than those of SM and FSM, which revealed 6.2% and 11.2% inhibition for TG, respectively. This observation was similar to previous findings [39] that have shown that omega-3 fatty acids decrease plasma triglyceride. This additional effect found in our study was likely due to the presence of omega-3 fatty acids, since one of the most noticeable effects of this fatty acid is a reduction in plasma triglycerides [40], possibly by decreasing triglyceride synthesis in the liver [41].

In the present study, haematological parameters Hb, MCH, and MCH in the DC group were significantly decreased compared to the NC group. Even though other parameters were also decreased, their differences were not significant. This observation agrees with Baskar et al.'s report (2006), which reported the effect of* Rubia cordifolia* in diabetic rats [42]. As a result of infections that occurred during diabetes, haematological parameters were reduced [43]. These parameters resulted in anaemic conditions when altered [44]. In diabetes mellitus, increased glycosylation of RBC membrane proteins causes anaemia [45] and it has been reported that lipid peroxides produced in this state lead to haemolysis of RBC [46].

Hb and RBC of the SM, FSM, and FSM + omega-3 groups and MCV and MCH of the SM and FSM + omega-3 groups were significantly increased compared to the DC group. These results were similar to the results of Ishimi et al. (1999), who showed that an intake of isoflavones in ovariectomized rats improved haematological parameters [47]. Soung et al. (2006) also observed that an intake of soy products in postmenopausal women had a beneficial effect on some haematological parameters [48] and that isoflavones may affect immune system functioning as a result of their receptors on lymphocytes [49].

In our study, a significant decrease in SOD activity and an increase in lipid oxidation (MDA level) were observed in diabetic control rats' blood. Elevated levels of lipid peroxides and the reduction of antioxidant enzymes have been reported to occur in the diabetic state [50]. In the hyperglycaemic state, sugars react with lipids and proteins that results in the generation of reactive oxygen species (ROS) [51]. This ROS enhanced lipid peroxidation [21].

In the current study, SM, FSM, and FSM + omega-3 groups showed significantly lower MDA and more SOD concentrations than the DC group. As was shown in our study, the antioxidant activities of soymilk and fermented soymilk have also been observed in other studies [52]. The antioxidative abilities of soymilk are related to soy isoflavones, soy protein, and saponins [53]. In our study, the significant effect of FSM products on the reduction of MDA when compared with the SM product was observed. This result showed that fermented soymilk had greater antioxidant and antimutagenic activities, relative to soymilk. An increase in the total antioxidant activity and antiradical effects of soymilk when fermented is also strongly supported by other studies [54].

In the present study, a significant decrease in MDA concentration was observed following administration of the FSM + omega-3 product when compared with SM and FSM products. In addition, with regard to SOD concentration, the FSM + omega-3 product showed higher SOD levels than did the SM and FSM products. These findings are supported by several researchers who have reported a decreased production in MDA concentration in human subjects treated with omega-3 fatty acids [55, 56].

The above findings further support the research of Barbosa et al. (2003), who found that omega-3 fatty acid supplementation may induce antiradical activities [57]. Omega-3 fatty acids can also prevent lipid peroxidation [58].

Our results showed that, compared with the DC group, SM showed lower hs-CRP concentrations, but this reduction was not significant. This may have been related to a low amount of soymilk intake in the current study. Soy components have contradictory results in terms of inflammatory parameters [59–61] and may be associated with lower levels of inflammatory parameters [62].

In our study, hs-CRP concentrations in the FSM + omega-3 group were significantly decreased compared with those of the DC and FSM groups. It appears that the significant effects observed in our study were related to omega-3. The anti-inflammatory activity of omega-3 fatty acids in many studies has previously been highlighted [63, 64]. The modulatory effects of omega-3 fatty acids on inflammation processes have also been found [63].

In summary, the present study showed that soymilk may be beneficial in reducing the risk of the onset of diabetes and in reducing the complications associated with diabetes, including the prevention of weight loss, lower blood glucose, plasma lipids, oxidative stress, and inflammation. The components of soymilk including soy protein, fibre, saponins, peptides, and particularly isoflavones are responsible for the effects observed in this research. In addition the efficiency of soy milk is increased with fermentation. The most obvious finding to emerge from this study was that omega-3 fatty acids can strengthen the effects observed. Generally, combining soymilk, probiotics, and omega-3 is effective for reducing complications associated with diabetes. Future studies on the current topic are therefore recommended.

## Figures and Tables

**Table 1:** Blood glucose change of each group fed with different products.

Group	Pre-intervention	Post-intervention						*P* value
		Week 1	Week 2	Week 3	Week 4	Week	Group	Week ∗ group
NC (*n* = 12)	114.08 ± 2.90^a^	110.75 ± 4.26^a^	111.00 ± 5.30^a^	103.30 ± 5.46^a^	133.33 ± 3.17^a^			
DC (*n* = 12)	162.58 ± 15.04^b^	172.17 ± 17.83^b^	206.92 ± 39.14^b^	222.42 ± 43.94^b^	223.75 ± 67.92^b^			
SM (*n* = 11)	153.45 ± 5.69^b^	149.73 ± 4.40^c^	144.18 ± 8.32^c^	145.55 ± 11.94^c^	143.64 ± 14.50^c^	>0/05	<0/001	<0/001
FSM (*n* = 11)	156.15 ± 7.42^b^	153.08 ± 6.13^c^	141.46 ± 8.02^c^	134.38 ± 14.62^dc^	133.92 ± 18.80^ac^			
FSM + omega-3 (*n* = 13)	160.45 ± 4.56^b^	157.27 ± 4.22^c^	136.91 ± 9.84^c^	127.09 ± 4.18d^ad^	120.36 ± 9.86^ac^			

NC = normal control, DC = diabetic control, SM = diabetic rats that received soymilk, FSM = diabetic rats that received fermented soymilk, FSM + omega-3 = diabetic rats that received fermented soymilk fortified with omega-3. Data are presented as mean ± SD. In each column, figures bearing different superscripts are significantly different at *P* < 0.05 (one way ANOVA with repeated measures).

**Table 2:** Body weights change of each group fed with different products.

Group	Pre-intervention	Post- intervention						*P* value
		Week 1	Week 2	Week 3	Week 4	Week	Group	Week ∗ group
NC (*n* = 12)	253.25 ± 23.53	270.08 ± 19.70^a^	270.83 ± 21.87^a^	284.50 ± 22.65^a^	299.67 ± 23.09^a^			
DC (*n* = 12)	245.67 ± 19.49	243.67 ± 20.27^b^	240.50 ± 20.92^b^	238.92 ± 21.06^b^	236.42 ± 21.19^b^			
SM (*n* = 11)	243.45 ± 17.41	255.09 ± 17.26^ab^	265.55 ± 17.73^a^	273.22 ± 19.58^a^	285.18 ± 19.45^a^	<0/001	<0/001	<0/001
FSM (*n* = 11)	250.54 ± 21.97	259.08 ± 24.92^ab^	272.23 ± 25.94^a^	283.62 ± 26.69^a^	295.15 ± 27.22^a^			
FSM + omega-3 (*n* = 13)	246.00 ± 19.54	256.36 ± 19.64^ab^	269.00 ± 18.94^a^	276.73 ± 21.05^a^	298.45 ± 21.84^a^			

NC = normal control, DC = diabetic control, SM = diabetic rats that received soymilk, FSM = diabetic rats that received fermented soymilk, FSM + omega-3= diabetic rats that received fermented soymilk fortified with omega-3. Data are presented as mean ± SD. In each column, figures bearing different superscripts are significantly different at *P* < 0.05 (one way ANOVA with repeated measures).

**Table 3:** Lipids profile of each group fed with different products.

Gorups	TC (mg/dL)	TG (mg/dL)	HDL-C (mg/dL)	LDL-C (mg/dL)
NC (*n* = 12)	50.50 ± 5.66^a^	52.50 ± 8.65^a^	38.83 ± 7.74	11.75 ± 2.41
DC (*n* = 12)	66.50 ± 15.32^b^	91.92 ± 33.33^b^	34.50 ± 16.82	11.83 ± 3.73
SM (*n* = 11)	53.91 ± 5.43^a^	59.45 ± 10.77^a^	41.55 ± 2.87	11.45 ± 2.73
FSM (*n* = 11)	56.62 ± 7.41^a^	62.77 ± 13.44^a^	43.08 ± 7.05	11.77 ± 2.38
FSM + omega-3 (*n* = 13)	52.64 ± 16.46^a^	55.73 ± 11.81^a^	39.64 ± 9.32	12.18 ± 2.48

NC = normal control, DC = diabetic control, SM = diabetic rats that received soymilk, FSM = diabetic rats that received fermented soymilk, FSM + omega-3 = diabetic rats that received fermented soymilk fortified with omega-3. Cholesterol, TG = Triglycerides, HDL-C = HDL cholesterol, LDL-C = LDL cholesterol. Data are presented as mean ± SD. In each column, figures bearing different superscripts are significantly different at *P* < 0.05 (one way ANOVA and Duncan test).

**Table 4:** Hematologic parameters of each group fed with different product.

Groups	WBC (×10^9^/L)	RBC (×10^12^/L)	PLT (×10^9^)	MCV (fl)	MCH (pg)	MCHC (g/dL)
NC (*n* = 7)	6.78 ± 1.67^ab^	7.96 ± .70^ab^	427.00 ± 180.66	52.60 ± 1.22^ab^	15.91 ± .48^a^	30.04 ± .99^a^
DC (*n* = 5)	5.60 ± 1.79^a^	7.25 ± .97^a^	489.80 ± 151.34	51.80 ± 1.94^a^	14.74 ± .62^b^	28.18 ± .74^b^
SM (*n* = 7)	7.88 ± 2.36^abc^	8.43 ± .52^b^	599.29 ± 65.28	54.70 ± 1.99^b^	15.77 ± .63^a^	28.95 ± .85^ab^
FSM (*n* = 7)	9.13 ± 1.91^bc^	8.20 ± .70^b^	514.00 ± 130.86	53.06 ± 2.79^ab^	15.31 ± 1.00^ab^	28.87 ± 1.23^ab^
FSM + omega-3 (*n* = 6)	9.52 ± 2.75^c^	8.90 ± .48^b^	522.20 ± 183.86	53.68 ± 2.19^b^	15.80 ± .98^a^	28.82 ± 1.07^b^

NC = normal control, DC = diabetic control, SM = diabetic rats that received soymilk, FSM = diabetic rats that received fermented soymilk, FSM + omega-3 = diabetic rats that received fermented soymilk fortified with omega-3.Data are presented as mean ± SD. WBC = white blood cells, RBC = red blood cells, Hb = hemoglobin, PLT = platelet count, MCV = mean corpuscular volume, MCH = mean corpuscular hemoglobin, MCHC = mean corpuscular hemoglobin concentration. In each column, figures bearing different superscripts are significantly different at *P* < 0.05 (one way ANOVA and Duncan test).
